# Enterococcus faecium, Staphylococcus aureus, Klebsiella pneumoniae, Acinetobacter baumannii, Pseudomonas aeruginosa, and Enterobacter (ESKAPE) Pathogens and Colistin Resistance in Ventilator-Associated Pneumonia: A Prospective Cross-Sectional Study

**DOI:** 10.7759/cureus.98097

**Published:** 2025-11-29

**Authors:** P B Praveen Kumar, Eunice Swarna Jacob, C K Bhuvaneswari

**Affiliations:** 1 Department of Microbiology, Thanjavur Medical College, Thanjavur, IND

**Keywords:** colistin resistance, eskape bacteria, mcr gene, mdr bacteria infection, ventilator-associated pneumonia (vap)

## Abstract

Ventilator-associated pneumonia (VAP) comes under hospital-acquired infections (HAI), which is an ongoing threat to both patients and healthcare workers. In our hospital, *Enterococcus faecium*, *Staphylococcus aureus*, *Klebsiella pneumoniae*, *Acinetobacter baumannii*, *Pseudomonas aeruginosa*, and Enterobacter* *(ESKAPE) pathogens play a major role in causing hospital-acquired infections. Out of 100 endotracheal aspirate samples, around 37% (n=37) of the isolates belonged to ESKAPE pathogens. However, only 12% (n=12) of the isolates were multidrug-resistant Gram-negative bacilli, and one isolate showed resistance to the last-resort drug, colistin (polymyxin E). Subsequently, we subjected that one isolate to conventional polymerase chain reaction, which detected the mobile colistin resistance-2 (MCR-2) gene. Unfortunately, by the time we found that isolate was colistin resistant, that patient had died due to multiple organ dysfunction syndrome (MODS). These findings underscore the need to perform antimicrobial susceptibility testing for all drugs according to our standard operating procedure (SOP) guidelines and conduct cascade reporting for all samples.

## Introduction

In 2008, a set of six organisms was grouped under the abbreviation "*Enterococcus faecium, Staphylococcus aureus, Klebsiella pneumoniae, Acinetobacter baumannii, Pseudomonas aeruginosa, and *Enterobacter (ESKAPE)," highlighting their global spread and resistance to the effects of antimicrobial medications [1]. The impact of antibiotic variety on ventilator-associated pneumonia caused by ESKAPE pathogens was reported by Sandiumenge et al. in 2011 [2].

Even though the beta-lactam antibiotic class is used across every aspect of medicine, colistin is still the last tool available to treat Gram-negative bacterial infections caused by multidrug-resistant isolates. However, 19 isolates of *Klebsiella pneumoniae* that caused ventilator-associated pneumonia (VAP) were shown to be resistant to colistin in a recent study conducted in Uttarakhand [3]. In our study, 37% (n=37) of the isolates belonged to the ESKAPE group of pathogens. But only one Gram-negative isolate showed resistance to colistin, which was detected by both phenotypic and genotypic methods.

This study was approved by the Tamil Nadu Dr. M.G.R. Medical University, Chennai, as a dissertation that was part of the postgraduate curriculum, as recommended by the National Medical Commission (NMC), India, and the Postgraduate Medical Education Regulations (PGMER) - 2023 [4].

## Materials and methods

Study design, place, and population

This study was conducted at Thanjavur Medical College, India, and was prospective and cross-sectional. The subjects of this study were symptomatic hospitalized adult patients who had been mechanically ventilated for the past 48 h and admitted between November 2023 and October 2024.

Sample collection

**Figure 1:**
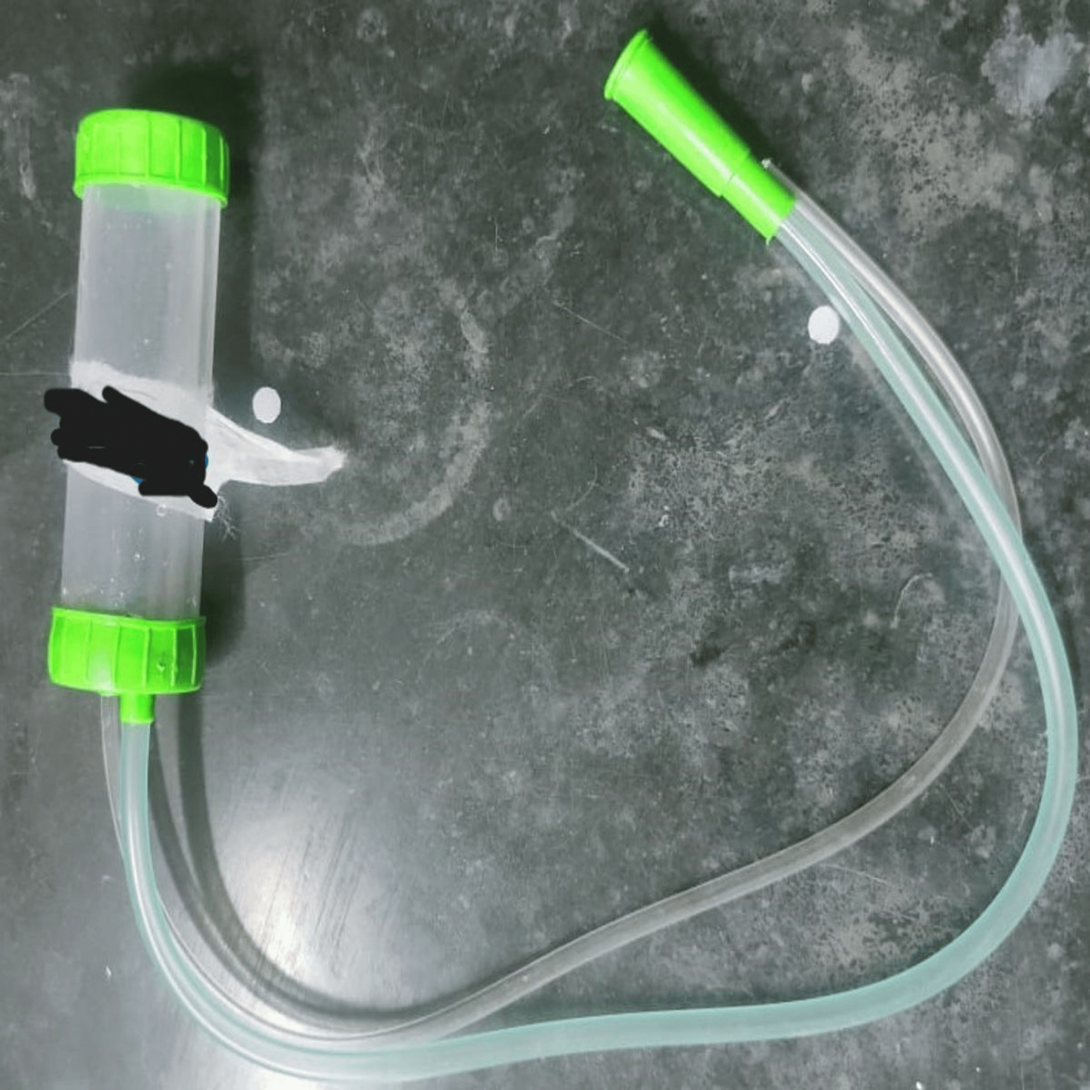
Lukens mucus trap.

In this study, endotracheal aspirate samples were collected from mechanically ventilated patients suspected of having ventilator-associated pneumonia in Lukens mucus trap (Figure 1).

Sample rejection criteria

Endotracheal aspirate samples received in an inappropriate container, incorrectly labeled or unlabeled samples, endotracheal swabs, and partial or full portions of endotracheal tubes were summarily rejected after discussing with the clinical team in an appropriate way.

Sample processing

**Figure 2:**
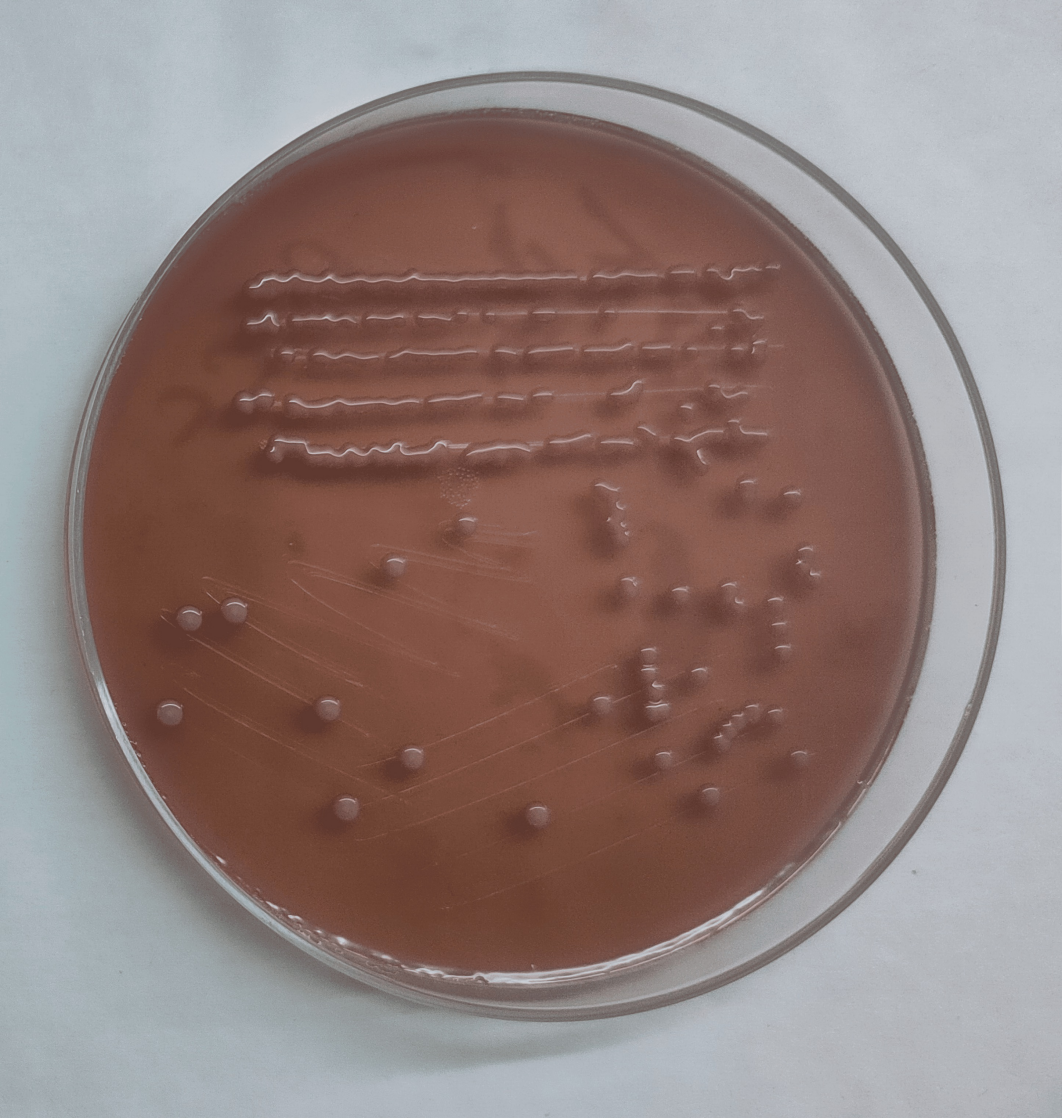
Semiquantitative streaking (quadrant streaking) method.

**Figure 3:**
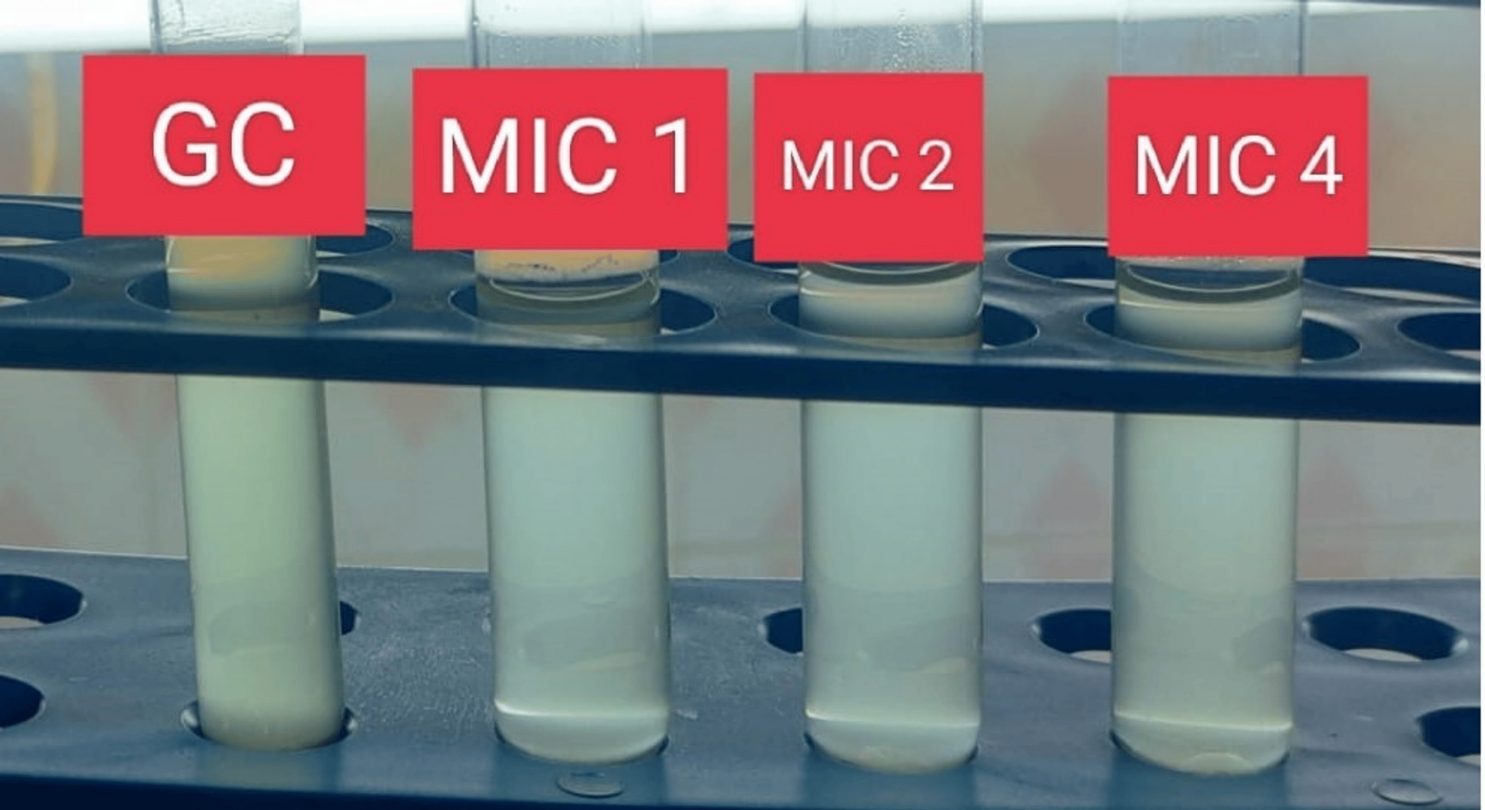
Phenotypic detection of colistin resistance using the colistin broth disk elution (CBDE) method. GC: growth control; MIC 1: minimum inhibitory concentration of 1 mcg/mL; MIC 2: minimum inhibitory concentration of 2 mcg/mL; MIC 4: minimum inhibitory concentration of 4 mcg/mL

**Figure 4:**
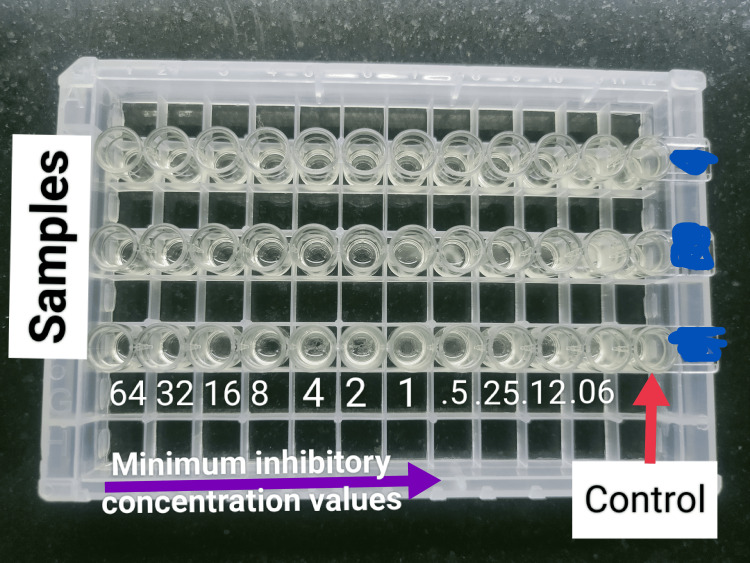
Phenotypic detection of colistin resistance using the colistin broth microdilution (BMD) method.

According to the grading formulated by Morris et al., all samples were subjected to a Gram stain, where the samples with less than 10 squamous epithelial cells and the presence of organisms were processed further for semiquantitative culture using the quadrant streaking method in a MacConkey agar plate and a blood agar plate, as shown in Figure 2 [5]. The plates were incubated aerobically at 37°C for 18-48 h. After 24-48 h of incubation, colony characteristics were observed and identified using conventional microbiological identification techniques. According to the Clinical Laboratory Standards Institute (CLSI) 2023 guidelines, antibiotic sensitivity testing (AST) was performed using the Kirby-Bauer disk diffusion method, and the results were interpreted accordingly [6]. Multidrug-resistant Gram-negative isolates were subjected to phenotypic and genotypic methods to detect colistin resistance (Figures 3, 4). Isolates detected as resistant to colistin by phenotypic methods were subjected to conventional polymerase chain reaction (PCR) followed by agar gel electrophoresis and viewed under an ultraviolet (UV) transilluminator for band observation.

Data analysis

The details of the culture-positive (n=39) samples were entered into a Microsoft Excel sheet (Redmond, WA: Microsoft Corporation). Later, we subjected these data to Fisher's exact test, and we compared the frequencies between the groups. A p-value less than 0.05 indicates statistical significance. In addition, we analyzed the antimicrobial resistance pattern of each isolate to know the current antimicrobial resistance trend in our location.

Ethical considerations

The study protocol was reviewed and approved by the Institutional Ethical Committee for Human Studies, Thanjavur Medical College, Thanjavur (approval number: 1183/2023). All participants provided informed consent prior to their inclusion in this study. Confidentiality of personal information was maintained at all parts of this study, and participants were informed of their right to withdraw from the study at any point of time without any compromise in the quality of their medical care.

## Results

**Figure 5:**
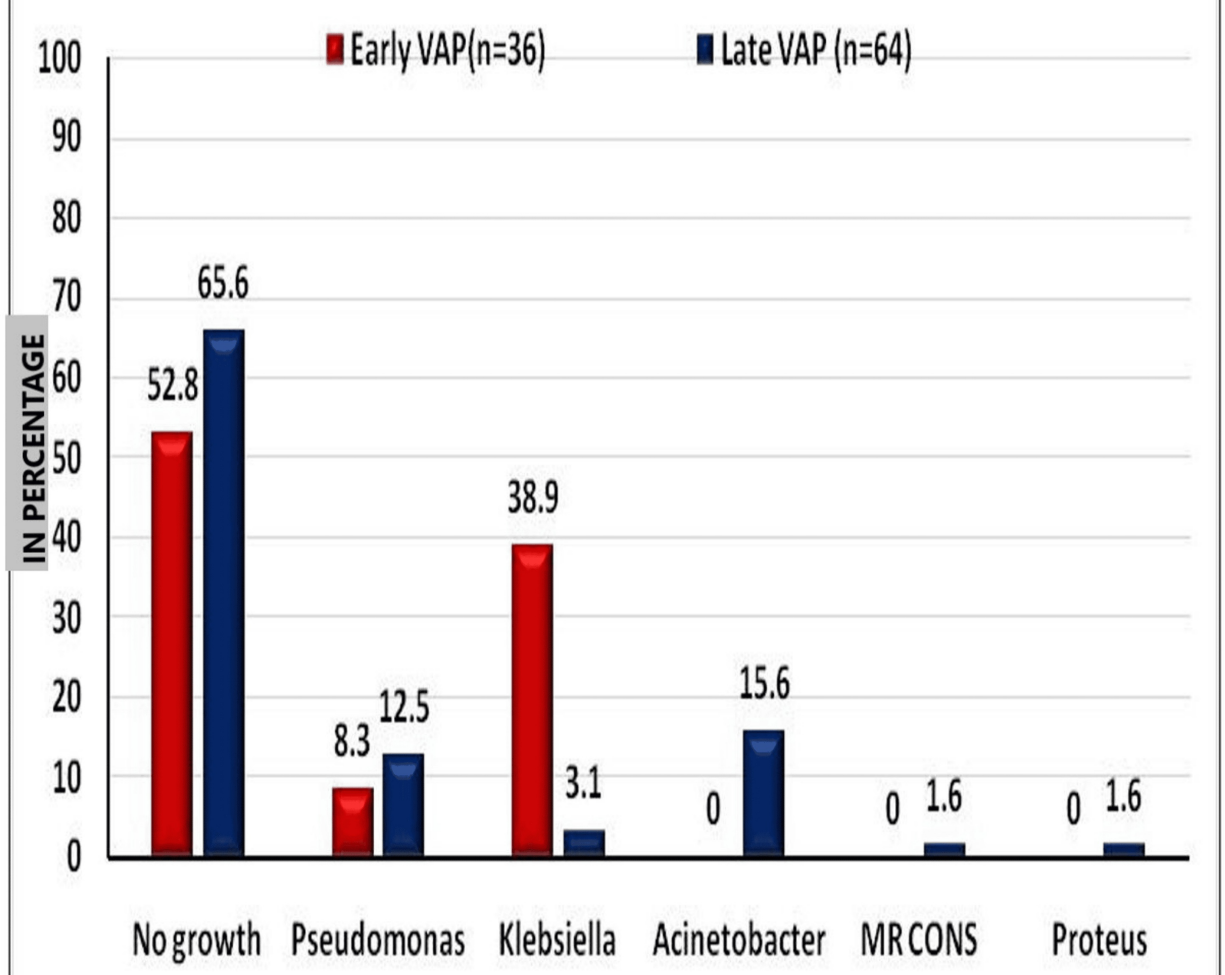
Graphical presentation of culture report analysis. VAP: ventilator-associated pneumonia; MR CONS: methicillin-resistant coagulase-negative staphylococci

Out of 100 endotracheal aspirate samples, 39 samples turned out to be positive for culture, and a p-value was calculated by Fisher's exact test (Table 1). Among the 39 culture-positive samples, except for two isolates, all other isolates belonged to the ESKAPE group of pathogens. Klebsiella species were found to be more common in early-onset VAP (number of ventilator days ≤4), whereas non-fermenters like Acinetobacter topped the list in late-onset VAP (number of ventilator days ≥5) (Figure 5).

**Table 1:** Culture report analysis of endotracheal aspirate samples (n=100). MR-SOSA: methicillin-resistant Staphylococcus other than *Staphylococcus aureus; *VAP: ventilator-associated pneumonia

Serial number	Culture report	Early onset VAP (n=36)		Late onset VAP (n=64)		Fisher’s exact test	Degrees of freedom (df)	p-Value
		n	%	n	%			
1	No growth	19	52.8	42	65.6	26.16	5	<0.0001
2	Pseudomonas	3	8.3	8	12.5			
3	Klebsiella	14	38.9	2	3.1			
4	Acinetobacter	0	0	10	15.6			
5	MR-SOSA	0	0	1	1.6			
6	Proteus	0	0	1	1.6			

Among the 38 Gram-negative isolates, only 12 isolates were multidrug-resistant. Subsequently, those 12 samples were subjected to the colistin disk elution method and the colistin broth microdilution method. Only one isolate showed resistance by means of both phenotypic methods. To confirm this further, that single isolate was tested genotypically, and the mobile colistin resistance-2 (MCR-2) gene was detected by conventional polymerase chain reaction.

## Discussion

Up to one-third of patients who need mechanical ventilation may develop ventilator-associated pneumonia (VAP), a serious clinical illness that has a high attributable morbidity and fatality rate [7]. Similarly, in the present study, colistin-resistant Klebsiella species were isolated in a sample. But unfortunately, that patient did not survive.

Among 100 study participants, confirmed VAP cases (both clinically and laboratory confirmed) were documented in 39 patients, accounting for nearly 5.80 per 1000 mechanical ventilator days, which was lower than the study conducted by Behera et al. in 2024 at Hyderabad with an incidence rate of 12.7 per 1000 mechanical ventilator days [8]. In India, although no national database is available, a 2014 multicentric study showed that the pooled VAP rate was 6.74 per 1000 mechanical ventilator days [9]. But our study showed the VAP rate was slightly lower than this.

In our study, the incidence of ESKAPE pathogens was found to be 37% (n=37), which was very much lower than that reported in a study conducted at a county emergency hospital in Romania in 2023 [10]. Similarly, in a study by Gupta et al. in Uttar Pradesh, 72.1% of isolates were identified to be multidrug-resistant Gram-negative pathogens [11]. But in our investigation, only 31.6% (n=12) of the isolates were multidrug-resistant Gram-negative isolates. In a study done in Greece, no difference was noted in the contribution of potentially multidrug-resistant pathogens [12]. But in our study, Klebsiella species were most common in early-onset VAP, while non-fermenters like Acinetobacter species were most common in late-onset VAP.

In Italy, one case was reported as colistin-resistant *Klebsiella pneumoniae* causing ventilator-associated pneumonia in a polytraumatic head injury patient [13]. In a similar manner, in this study, colistin-resistant Klebsiella species were isolated from the patient admitted to the intensive care unit after he met with a road traffic accident with head injuries. In a study done at the National Research Institute of Tuberculosis and Lung Diseases (NRITD) in Iran, the MCR-1 gene was not detected [14]. Similarly, in our study, the MCR-1 gene was not detected. But the MCR-2 gene was detected for only that one colistin-resistant isolate. So, to prevent morbidity and mortality due to VAP, bundle care maintenance plays a vital role in preventing infectious complications in patients who are all on mechanical ventilation.

Limitations and strengths

One of the main limitations of the study is the failure to report the bundle care adherence rate at our hospital. Additionally, not all antimicrobial agents mentioned in the Clinical Laboratory Standards Institute (CLSI) 2023 guidelines were tested [6]. It may affect the completion of the antimicrobial resistance profile. In Tamil Nadu, this work is the first to reveal a colistin-resistant bacterium causing ventilator-associated pneumonia, using both phenotypic and genotypic approaches.

## Conclusions

The emergence of such multidrug-resistant ESKAPE pathogens causing ventilator-associated pneumonia (VAP), compounded by the lack of availability of new antimicrobials, provides a warning that a grave future is waiting for the management of healthcare-associated infections. Therefore, it is important to frame local policies and measures and to take affirmative actions to prevent healthcare-associated infections and reduce the burden of multidrug resistance.

Although colistin is the drug of choice for carbapenem-resistant bacteria, the emergence of colistin resistance poses a significant challenge. Hence, innovative therapies are advocated. It is also important to emphasize the need for proper antimicrobial drug usage and to prevent the emergence of multidrug-resistant strains.
